# Template of a climate sustainability plan for medical professional organizations: the Canadian Association of Gastroenterology example

**DOI:** 10.1093/jcag/gwae051

**Published:** 2024-12-21

**Authors:** Desmond Leddin, Paul Sinclair, Harminder Singh, Rachael Sherman

**Affiliations:** Division of Digestive Care and Endoscopy, Department of Medicine Dalhousie University, Victoria General Hospital, Halifax, NS B3H2Y9, Canada; INSINC Consulting Inc., Guelph, ON N1G 3G3, Canada; Section of Gastroenterology, Department of Internal Medicine, Max Rady College of Medicine, University of Manitoba, Winnipeg, MB R3E 3P4, Canada; The Estée Lauder Companies, New York, NY 10153, United States

**Keywords:** sustainability plan, environmental change, climate change, medical organizations

## Abstract

Environmental change is underway and has the potential to adversely affect digestive health. Professional medical organizations have an important role to play in addressing the challenge. An important initial response is the development of a sustainability plan for the medical organization. There are no standardized criteria as to what should be included in such a plan. We have proposed 12 key components that should be contained in sustainability plans for medical organizations. We describe how these were developed for the Canadian Association of Gastroenterology (CAG) and plans for future implementation. We hope that the CAG plan may serve as a template to assist peer medical organizations optimize their response to the climate crisis.

## Objectives

The purpose of this paper is to describe the climate sustainability plan of the Canadian Association of Gastroenterology (CAG) and to suggest key components that may be used to develop sustainability plans for medical professional organizations.

## Background

Environmental change, which includes global warming, pollution, and biodiversity loss is adversely affecting health.^1^ Health care, paradoxically, is contributing to the problem through its generation of greenhouse gases and waste, such as plastics.^2^

Medical professional organizations, such as the CAG are important in facilitating and promoting the professional activities of their memberships. As the climate crisis evolves, they have an important role to play in meeting the challenge that environmental change poses for health and healthcare delivery.

Developing a sustainability plan for an organization is an important step toward addressing environmental change. Having a plan can help ensure that the organization mounts an effective response, that the objectives are clear to all members, and can signal concern to the government and the public.

Although sustainability plans are common in business organizations,^3^ they are still relatively uncommon in medical professional organizations. Medical professional organizations, like the CAG, are not standard commercial businesses. Most are not-for-profit and are not involved in the manufacturing or shipping of products. Currently, there are no guidelines, which outline what might be considered the key components of a sustainability plan for medical professional organizations. Consequently, those plans which have been published vary widely in format and content. By describing the process and content of the CAG sustainability plan, we wish to share a template that may be useful to other medical professional organizations.

### Process and preparation

In 2023 the CAG Board of Directors approved the formation of a Climate Committee. The committee was charged with developing a sustainability plan for the organization (see Supplementary File 1 for the completed plan and Supplementary File 2 for the Terms of Reference of the Climate Committee). We were fortunate to secure the support and guidance of an individual with professional experience (RS) in developing sustainability plans for major business companies.

Input on the plan was obtained from leadership, Chairs, and Co-chairs of all CAG committees attending an annual strategic retreat. A survey was sent to attendees prior to the retreat (Supplementary File 3). The survey explored questions such as how much of a priority this issue was for each committee, the concerns that individual Chairs felt on this issue, how the Chairs felt their committee could engage, perceived barriers to engagement, where this issue should be on the strategic priorities of the CAG and achievable objectives. At the retreat, a half day was allocated to the topic. Following presentations on climate and health and a review of the survey results, small groups were convened and reported back to the plenary session on the development of the plan. Using this input, and taking elements from industry plans, key components were identified as shown in Table 1. The total time allocated to the development of the plan, including the contributions of multiple individuals, is difficult to estimate precisely but was likely in the range of 80-100 hours.

**Table 1.** Key components of Sustainability Plans for professional medical organizations.

Structure of the Climate Committee and terms of reference Scope of the plan Vision Mission Reduction of greenhouse gases proposal Research Education Communication strategy Resilience and adaptation Engagement and Advocacy Finances Monitoring and adjustment

The following briefly expands on the key components.

- The structure of the organization’s climate committee, its terms of reference, and how it fits into the overall governance structure, should be described.
- The scope of the sustainability plan describes the stakeholders to whom the plan applies. The scope could be internal and confined to the organization (emissions scopes 1 and 2), external focused on key partners (emissions scope 3), or a combination of both.
- The vision is where the organization sees itself on this issue in the future.
- The mission describes the current activities of the organization on sustainability and is intricately linked to the general objectives.
- A statement on how the organization intends to reduce its greenhouse gas emissions, which are at the core of global warming, is desirable. What is meant by sustainability, which can have many different meanings, should also be described. Some organizations may aim for net zero or align with a pathway based on the Paris Agreement, current climate science carbon neutrality, or simply reduce emissions as much as possible while relying on other sectors of the healthcare system to achieve net zero. For many medical professional organizations, the direct greenhouse emissions associated with running the organization are very small and the major contribution to emissions will be from the annual meeting and other meetings of the organization’s Board and Committees^4^ and due primarily to transportation. The organization may wish to state how these will be reduced, and to what extent.
- Research and education on environmental change are core to the mission as is communication of best practices. How these aspects of the mission will be executed should be described.
- Building resilience and adaptation is required because, regardless of current decreases in greenhouse gas emissions, the climate will continue to warm for decades to come. This will have a wide-ranging impact on patients, healthcare professionals, and communities.
- Engaging with national and international partners and being effective advocates for change are core professional values.
- Some of the initiatives on which organizations can engage, such as measuring the carbon footprint of members’ professional activities such as endoscopy, and other care-related activities require funding. Seeking out sources of funding can be part of the mission.
- Some organizations may wish to include a statement on their divestment from fossil fuel investments.
- Monitoring of objectives realized, and ongoing readjustment of the plan is an important part of ensuring that progress is achieved.

Specific objectives, and timelines for reaching the objectives, are then developed within this framework in discussion with the leads of each of the organization’s committees. The specific objectives will vary by committee, depending on the emphasis and importance of each of the general objectives to the committee. For example, advocacy may not be a key objective of the endoscopy committee, but education of the organization’s membership and research on the footprint of endoscopy likely will be key components of the endoscopy committee’s activities.

### Moving to action

The CAG Sustainability Plan was designed to be launched in 2 phases.

Phase 1: The first 12 months of the plan were considered the foundation year with the focus on setting up the governance structure, such as terms of reference of the climate committee, reaching some readily achievable goals, but primarily the focus was on raising awareness and educating the membership and leadership, and other partners including the public.

Phase 2: The second phase, which is under development, will involve working with, and developing, objectives for each of the organization’s committees. It is recognized that some of these will wish to engage more, or more rapidly, than others and priority will be given to those committees who are ready to engage on the issue.

### Future

Progress has already been made in measuring the travel-related carbon footprint of the annual meeting, preparing education materials for members, patients, and the public, and several research projects that will be of benefit to both members and the wider community. The next steps include a comprehensive measurement of the footprint of the CAG annual meeting and developing targets for each of the CAG committees. Having a comprehensive sustainability plan provides the framework to address the challenge of environmental change. We hope what we consider to be core components listed in Table 1 will be useful to other medical organizations wishing to develop their own plans.

## Supplementary data

Supplementary data are available at *Journal of the Canadian Association of Gastroenterology* online.

gwae051_suppl_Supplementary_Materials

## Data Availability

No new data were generated or analysed in support of this manuscript.
