# Supplementary material for: Template of a climate sustainability plan for medical professional organizations: the Canadian Association of Gastroenterology example
Source: J Can Assoc Gastroenterol. 2024 Dec 21;8(1):4–6. doi: 10.1093/jcag/gwae051 (PMC11788559; doi:10.1093/jcag/gwae051)
Supplement: gwae051_suppl_Supplementary_Materials [file gwae051_suppl_supplementary_materials.zip › gwae051_suppl_Supplementary_Files_1.pdf]

Canadian Association of Gastroenterology

Climate Sustainability Plan.

This document has been altered from the original to remove appendices which are not relevant to a wider readership and includes descriptions of CAG committees to allow a better understanding of the structure of the organisation.

Summary: Climate change is increasingly recognized as a public health challenge with major implications for digestive health. Paradoxically healthcare contributes to the problem by generating greenhouse gases, and pollution. Healthcare related environmental harm cannot be eliminated but we have an ethical duty to minimize it as much as possible. In response to these challenges, the board of the Canadian Association of Gastroenterology approved the formation of a climate committee. One of the first actions of the committee was to develop a draft sustainability plan for the organization. This draft plan has been developed by the committee at large with the support of an external sustainability expert. The plan outlines the rationale behind why the Association should act, what the benefits might be to the organization, how action could be taken, the vision and mission of CAGs climate plan, and the next steps if the board wishes to proceed. The carbon footprint directly attributable to the organization is very small, however CAG is in a position to influence individuals, organizations, and health systems with whom we collaborate. CAG can be a contributor to systems wide change. The plan outlines how this may be done starting with a foundation year in 2024 in which the leadership of the organization is made more aware of the problem and educated on the issues. It is hoped that post 2024 it may be possible to deliver a specific set of actions through each of the CAG committees, depending on the degree of relevance of the issue to them, and their eagerness to participate.

Background and rationale.

In 2023 the Canadian Association of Gastroenterology established a climate change committee. One of the objectives of the committee is to produce a climate sustainability plan for the organization.

Sustainability broadly is “meeting the needs of the present without compromising the ability of future generations to meet their own needs”. (5) Sustainability consists of environmental, social, and economic factors. Sustainability covers topics including human rights, waste, water, climate, and several more as outlined by the United Nations Sustainable Development Goals. The focus of this document is specifically climate sustainability.

The rationale behind the development of a climate sustainability plan is that the climate crisis is now recognized as a public health emergency.(6) Paradoxically healthcare is a significant contributor to the problem in that it produces about 5% of total global greenhouse gases and is a significant contributor to pollution (7)

There are many reasons why moving to sustainable practice makes sense.(5) It is in our self-interest, it is in society’s interest, it may make economic sense, and it is ethical. As healthcare providers we have an obligation to not harm patients. We know that healthcare related emissions and pollution are contributing to patient harm. It therefore follows that we have an ethical obligation to reduce healthcare missions as much as possible. The sustainability plan helps move CAG to that position.

In the broadest sense, sustainability means conducting the operations of today in such a way that it does not compromise the needs of future generations. The focus of climate sustainability is to help ensure that the planet stays on track to keep global temperature rise below 1.5°C. This includes helping the organizations with whom CAG interacts moving to the same goal.

CAG’s climate sustainability can serve several purposes. It can contribute to the discussion on building resiliency. Increasing frequency of storms, weather events and infrastructure threats is likely as the planet warms and will affect delivery of health care. One way to reduce the impact of this is to increase the resiliency of healthcare systems and supply chains. The emissions under direct control of the organization are very small however we are in a position to influence emissions from the wider healthcare system, which could be

very significant. Having a sustainability plan will enhance the reputation of the organization. The climate crisis is going to continue to evolve. By having a plan in place, we will position ourselves to be able to contribute to solutions. While it is still early in the climate change response, this will become increasingly important, and it will be especially important to younger potential new members of the association. To maintain a relevance in this area it is important that we have a plan to show that we are moving forward. By engaging in this area, we encourage innovation and promote research and education on the topic. Stakeholder engagement is important and this issue will become increasingly relevant to them. Our stakeholders include not only members of the Association, but those whom we influence in the health system, and the public by means of our work in the Canadian digestive health foundation.

Structure of CAG

CAG is a self-governing, not for profit, organization of healthcare providers, and industry, focussed on digestive care. It is directed by a board and a chief executive officer. An operations committee is responsible for operationalizing the policies of the board. There are six portfolios, each (except for nominations) containing numerous committees. (Table 1). The function of most of the committees is obvious from its name. Regional reps refers to regional representation from provincial associations. GRIT refers to the Gastroenterology Residents in Training program. MOC is Maintenance of Certification program from the Royal College of Canada. Scholars is a program designed to encourage internal medicine residents to pursue gastroenterology as a career. SEE is the Skills Enhancement program for Endoscopy.CGRS refers to the Canada Global Rating Scale for quality improvement in endoscopy. IBD GRS is a quality improvent tool for IBD. The strategic objectives of association are carried out by the portfolio and committee structure.

Portfolio	Committees					
**Administration**	Admissions	Diversity	Ethics	Publications	GIWomen Can	Women's Mentorship	Advocacy
**Clinical**	Endoscopy	Pediatrics	Practice Affairs	Regional Reps			
**Education**	GRIT	MOC	Program Directors	Scholars	SEE	Trainee	
**Quality**	Innovation	Quality Practice	CGRS	IBD GRS	Reporting	Choosing Wisely	
**Research Affairs**	Research Topics	Trainees					
**Nominations**							

Table 1. The structure of the Canadian Association of gastroenterology showing portfolio and committees.

Process

The project was discussed by the Canadian Association of Gastroenterology climate committee, and a subgroup was set up to work on this, supported by a sustainability expert. The issue was the focus at the leadership forum in Toronto at the end of October 2023, where a half-day was devoted to the issue of climate change. We were able to get the input of many portfolio leads and committee members.

Scope of the plan.

The scope or reach of the plan is directed both internally and externally. Internally it is hoped that the plan will guide the operations of the head office and the core committees as outlined in Table 1. Externally, it is directed towards the membership at large, the regional gastroenterology organizations, health care providers in other disciplines, regional and federal governments and vendors who supply goods and services to CAG and health care providers in our networks. There is also an international element to this in the CAG does influence the behaviour of other organizations globally.

There are many different greenhouse gases. Organizations, businesses, and individuals generate these through three scopes, or routes. (8)

- Scope 1 emissions are greenhouse gases, which are **generated by company, facilities, or company vehicles**. In the case of the Canadian Association this would be primarily the emissions associated with the head office, which are likely near zero in that the office does not produce greenhouse gases directly.
- Scope 2 emissions are indirect greenhouse gas emissions, associated with the **purchase of electricity, water, steam, heat and cooling and waste disposal.** Although scope 2 emissions physically occur at the facility where they generated, they are accounted for in an organizations inventory because they are result of the organization's energy use.
- Scope 3 emissions are the result of activities from assets **not under control by CAG, but which CAG indirectly affects**. Scope 3 emissions include all sources not within CAG's scope 1 one and Scope 2 boundary. For example, the CAG holds annual meetings at a conference centre or in a hotel. This indirectly results in carbon emissions, which the organization does not control. Employee travel is also included under Scope 3.

The CAG has direct control over only a small proportion of the emissions with which it is associated (Table 2). The CAG can control the environment, to an extent, of the national office, it can influence how meetings of sub committees are held, and it can influence the conduct of the annual meeting. CAG does not own the premises or convention centres where these meetings are held. It can, however, control the location of the meeting which has an important impact on associated emissions, and it can choose the hotels and convention centers, which are committed to sustainability.

**Influence**	
**Direct (Scope 2 Emissions)**	**Indirect (Scope 3 Emissions)**
Head office of the CAG.<br>Head office of CDHF<br>Meetings of committees (online versus in person)	Annual meeting of organisation<br>Suppliers<br>Membership practice such as endoscopy<br>Regional GI Associations<br>Royal College<br>CMA<br>Provincial and Federal Government<br>Global Gastroenterology

Table 2. Operations which CAG can affect either directly or indirectly and attributable scope, two and three emissions.

Vision

The Canadian Association of Gastroenterology will become a model of a healthcare organization which practises sustainably, incorporates sustainability into its operations and decisions and which facilitates the groups whom it influences moving to a more climate friendly, low carbon, low emission practice.

Mission and General Objectives

The climate sustainability plan of the CAG will reflect the mission objectives of the organisation and that of the climate committee which are;

- Reduce greenhouse emissions.
- Engage in research on the impacts of climate change from a digestive health provider perspective.
- Educate the GI community including industry and the public on the implications of environmental change on digestive health.
- Communicate best practices to mitigate the impact on climate.
- Build resilience and adaptation.
- Work with and leverage national and international partners.
- Recommend advocacy opportunities for the CAG to engage government decision-makers.
- Make recommendations on sustainable funding sources to support the work of the Committee.

Moving to Action

The ultimate goal is that the principles of sustainability will be woven into the fabric of all of the CAG committees. The amount of action any individual committee might take will be determined by the relevance to the committee, and by the interest of the committee members. Work needs to be done on education and raising awareness of both the membership and the committee leadership of the organization.

Foundation year - 2024

2024 will be the foundation year. The focus will be on raising awareness and educating four groups.

- CAG leadership, committee leads, and committee membership.
- Regional GI associations
- Membership at large
- Public

Subsequent

Following the actions of the foundation year on building awareness and education, it is hoped that in subsequent years it will be possible to fill in more specific objectives for the rest of the organization's committees.

Monitoring and Evaluation.

This sustainability plan is very much intended to be a work in progress, a process, rather than a fixed plan. The plan will need to be adjusted based on results and feedback. The intention would be to do this on a continuing basis and submit a review to CAG's governing board annually.

Acknowledgments.

The committee wishes to acknowledge the contribution of our external sustainability plan expert, Rachael Sherman for her advice and guidance, that of the committee members who participated in the work, and the support of Angela Siracusa from the CAG office.

Desmond Leddin and Harminder Singh: Committee co-chairs.

John Igoe. Frances Tse, Grigoris Leontiadis, David Armstrong, Paul Sinclair, Dan Sadowsky, Cara Pray, Russell Yanofsky, Ciaran Galts, Neal Shahidi and Kelsey Cheyne (CDHF).
