# Supplementary material for: Template of a climate sustainability plan for medical professional organizations: the Canadian Association of Gastroenterology example
Source: J Can Assoc Gastroenterol. 2024 Dec 21;8(1):4–6. doi: 10.1093/jcag/gwae051 (PMC11788559; doi:10.1093/jcag/gwae051)
Supplement: gwae051_suppl_Supplementary_Materials [file gwae051_suppl_supplementary_materials.zip › gwae051_suppl_Supplementary_Files_2.pdf]

CAG Climate Change Committee

Terms of Reference

Purpose

The Canadian Association of Gastroenterology (CAG), under the leadership of CAG Administrative Affairs, is committed to providing education and engaging in research on the impact which environmental change can have on digestive health, and on how delivery of digestive care impact environmental health, and to offer solutions to these issues.

The primary target audience includes clinicians, researchers and health care administrators in gastroenterology/hepatology and trainees entering the practice of gastroenterology.

Leadership and Composition

Composition of the Committee will follow the guidelines contained in the CAG Governance Policies:

- Volunteers must be CAG members in good standing.
- Where possible, the individuals should have relevant interest and/or experience in the subject matter/mandate of the Board of Directors/committees. *In exceptional circumstances, where individuals may not have the subject-matter expertise, non members may be appointed to a committee or task force by, and at the discretion of, the relevant Vice President. Such individuals may not act in a leadership role.*
- Potential volunteers must have the time and capacity to be engaged during the term of their appointment. Diversity, Equity, and Inclusion: The core principles of diversity, equity and inclusion must, and shall be, considered for volunteer recruitment and engagement throughout the CAG. The CAG should strive to ensure that volunteers reflect the broad communities which we represent. The CAG must continually, and actively, engage and include all members regardless of their race, ethnicity, culture, age, gender, sexual orientation, gender identity and ability. Further, the CAG must be proactive to ensure that underrepresented communities are engaged throughout the CAG, and such diversity is reflected in our volunteer population.
- Geographic diversity – as a national organization it is imperative that committees comprise a strong geographic diversity within their respective populations. Further, this geographic diversity shall be extended to the volunteer leadership as well (directors, vice presidents, chairs/co-chairs/vice chairs).
- Succession planning – committee volunteers should be considered for 'advancement' to chair/co-chair/vice-chair, vice president and director roles. The CAG encourages a 'promote from within' approach.

The CAG Climate Change Committee is led by two Co-Chairs.

The Committee comprises clinical and basic researchers and clinicians. There must be at least two Trainee members. A Canadian Health Digestion Foundation (CDHF) representative will also be a member of the Committee.

A Steering Committee comprising the Co-Chairs, and up to three Committee members, may be formed as needed and will act as the 'Executive Committee', with input and direction from the broader Committee. The Steering Committee may attend and participate in the annual Fall Forum on behalf of the Committee.

The Committee will comprise up to 12 members. All members of the Committee (except the CDHF representative) must be CAG members in good standing.

Terms

The term of Committee members will be two years, with a renewal of one two-year term.

Reporting Structure

The Climate Change Committee reports to the VP, Administrative Affairs.

Through the VP, Administrative Affairs, the Climate Change Committee will engage and dialogue with the Operations Committee on a regular basis, to ensure a feedback and input loop with the five Affairs groups.

The Climate Change Committee is a research, education and recommending body. All final decisions on policy and execution will be made by the Board of Directors.

Funding

The Committee Co-Chairs will work with their committee and the VP, Administrative Affairs, on an annual basis to submit budget requests to the Board of Directors. The Committee is encouraged to make recommendations on sustainable funding sources to help support its work.

The expectation is that Committee members must participate actively in online and in-person meetings. Membership is contingent on engaging in at least 50% of the meetings.

Strategic Objectives

1. Research
   To engage in research on the impacts of climate change from a digestive health provider perspective
2. Education
   To educate the GI community including industry and the public, on the implications of environmental change on digestive health.
   To communicate best practices to mitigate the impact on climate, build resilience and adaptation.
3. Build networks.

To work with and leverage national and international partners on this work.

4. Advocacy
   To recommend advocacy opportunities for the CAG to engage government decision-makers.
5. Sustainability.
   To develop a sustainability plan for the CAG
6. Secure funding
   To make recommendations on sustainable funding sources to support the work of the Committee.
