# Supplementary material for: Template of a climate sustainability plan for medical professional organizations: the Canadian Association of Gastroenterology example
Source: J Can Assoc Gastroenterol. 2024 Dec 21;8(1):4–6. doi: 10.1093/jcag/gwae051 (PMC11788559; doi:10.1093/jcag/gwae051)
Supplement: gwae051_suppl_Supplementary_Materials [file gwae051_suppl_supplementary_materials.zip › gwae051_suppl_Supplementary_Files_3.pdf]

CAG Climate - Leadership Survey

Start of Block: Default Question Block

Q1 What is your role in the CAG?

- ○ Member of executive (1)
- ○ Portfolio chair (2)
- ○ Committee chair or member (3)
- ○ Admin support (4)
- ○ Prefer not to say (5)

Q2 Please indicate which committee or committees you serve on. If you are a portfolio chair click all the committees in your portfolio.

- ☐ Diversity, equity, inclusion (1)
- ☐ Ethics (2)
- ☐ Publication and archives (3)
- ☐ Admissions (4)
- ☐ GI Women CAN (5)
- ☐ Women's mentorship program (6)
- ☐ Endoscopy (7)
- ☐ Paediatrics (8)
- ☐ Practice affairs (9)
- ☐ CDDW planning (10)
- ☐ GRIT (11)
- ☐ SEE (12)
- ☐ Innovation (13)
- ☐ Practice audit (14)
- ☐ Choosing Wisely (15)

- [ ] CCGRS (16)
- [ ] IBD GRS (17)
- [ ] Clinical research (18)
- [ ] Basic science research (19)
- [ ] Regional Representation (20)

Page Break

Q3 How much of a priority do you think the issue of climate change should be for CAG?

	None at all	A little	A moderate amount	A lot	A great deal
	0 10	20 30	40 50 60	70 80	90 100
Move the slider from 0= least priority to 100 = most important priority ()					

Page Break

Q4 Considering both your personal and professional life how concerned are you about climate change?

	0 10 20 30 40 50 60 70 80 90 100
Move the slider from 0= not concerned at all to 100 = I think this is a major crisis ()	

Page Break

Q5 With regard to climate change and your committee please indicate your agreement, or disagreement, with the following statements.

	Strongly disagree (1)	Disagree (2)	Neutral (3)	Agree (4)	Strongly agree (5)
All CAG committees need to engage on this issue (1)	○	○	○	○	○
I do not think it is an issue for my committee (2)	○	○	○	○	○
My committee would like to do something (3)	○	○	○	○	○
I have clear ideas about what my committee might do. (4)	○	○	○	○	○

Q6 With regard to barriers to engagement on this topic please indicate your level of agreement.

	Strongly disagree (1)	Disagree (2)	Neutral (3)	Agree (4)	Strongly agree (5)
My committee has more pressing priorities. (1)	☐	☐	☐	☐	☐
This topic is outside my committee's focus. (2)	☐	☐	☐	☐	☐
This is an issue which government, rather then CAG, needs to handle. (3)	☐	☐	☐	☐	☐
Unlikley we can make a difference. (4)	☐	☐	☐	☐	☐
No barriers, we are good to go. (5)	☐	☐	☐	☐	☐
We do not have the knowledge base to engage. (6)	☐	☐	☐	☐	☐

Q7 What do you think the strategic goals for CAG as a whole should be on climate change?

____

____

____

____

____

Q8 Can you think of any SMART goals (specific, measurable, achievable, realistic, timely) for your committee around the issue of climate?

____

____

____

____

____

Page Break

Q9 Do you have any suggestions with regard to supports which might help your group move ahead, such as more education, slide decks, speakers, meeting with an expert?

____

____

____

____

____

Q10 Any other thoughts, suggestions?

____

____

____

____

____

End of Block: Default Question Block
